# An International Multicenter Performance Analysis of Cytomegalovirus Load Tests

**DOI:** 10.1093/cid/cis900

**Published:** 2012-10-24

**Authors:** Hans H. Hirsch, Irmeli Lautenschlager, Benjamin A. Pinsky, Laura Cardeñoso, Shagufta Aslam, Bryan Cobb, Regis A. Vilchez, Alexandra Valsamakis

**Affiliations:** 1 Transplantation and Clinical Virology, Department Biomedicine (Haus Petersplatz), University of Basel, Switzerland; 2 Department of Virology, Helsinki University Hospital and University of Helsinki, Finland; 3 Department of Pathology, Stanford University, Palo Alto, California; 4 Department of Microbiology, Hospital Universitario de la Princesa, Madrid, Spain; 5 Roche Molecular Systems, Inc, Pleasanton, California; 6 Department of Pathology, The John Hopkins Hospital, Baltimore, Maryland

**Keywords:** CMV, viral load, PCR, transplantation, standardization

## Abstract

***Background.*** Quantification of cytomegalovirus (CMV) load is central to the management of CMV infections in immunocompromised patients, but quantitative results currently differ significantly across methods and laboratories.

***Methods.*** The COBAS AmpliPrep/COBAS TaqMan CMV Test (CAP/CTM CMV test), developed using the first World Health Organization CMV standard in the calibration process, was compared to local assays used by 5 laboratories at transplant centers in the United States and Europe. Blinded plasma panels (n = 90) spiked with 2.18–6.7 log_10_ copies/mL and clinical plasma samples from immunocompromised patients (n = 660) were tested.

***Results.*** Observed mean panel member concentrations by site and 95% confidence intervals (CIs) of the data combined across sites were narrower for CAP/CTM CMV test compared with local assays. The 95% CI in log_10_ copies/mL of the combined data per panel member for CAP/CTM CMV test vs comparator assays was .17 vs 1.5 at 2.18 log_10_ copies/mL; .14 vs .52 at 2.74 log_10_ copies/mL; .16 vs .6 at 3.3 log_10_ copies/mL; .2 vs 1.11 at 4.3 log_10_ copies/mL; .21 vs 1.13 at 4.7 log_10_ copies/mL; and .18 vs 1.4 at 6.7 log_10_ copies/mL. In clinical specimens, constant and variable quantification differences between the CAP/CTM CMV test and comparator assays were observed.

***Conclusions.*** High interlaboratory agreement and precision of CAP/CTM CMV test results across 5 different laboratories over 4 orders of magnitude suggest that this assay could be valuable in prospective studies identifying clinical viral load thresholds for CMV treatment.

## Abstract (precis)

A new quantitative polymerase chain reaction assay, COBAS AmpliPrep/COBAS TaqMan CMV Test, was developed using the first World Health Organization cytomegalovirus standard. It demonstrated a high level of interlaboratory agreement and precision compared to quantitative results obtained with tests used by 5 different laboratories.

**(See the Editorial Commentary by Caliendo on pages 374–5.)**

Human cytomegalovirus (CMV) infection causes significant morbidity and mortality in the posttransplant period of both solid organ transplant (SOT) and hematopoietic stem cell transplant (HSCT) recipients [1–5]. Seronegative recipients of transplants from seropositive donors are infected by CMV transmitted through the transplanted organ or inadvertently through CMV-positive blood products [1–5]. In CMV-seropositive recipients, reactivation of latent CMV occurs when CMV-specific immune control is impaired by immunosuppressive drugs and T-cell–depleting therapies [1–5].

The diagnosis of CMV replication and disease in SOT and HSCT recipients can be made using different laboratory methods, including histology, pp65 antigenemia, or CMV DNA by quantitative nucleic acid testing (QNAT) [1–5]. Culture methods of body fluids and tissue samples are generally slow and are not quantitative [4]. The pp65 antigenemia test is rapid (1 day time-to-result) but less sensitive than QNAT and often difficult to perform on severely neutropenic patients [1–7]. CMV QNAT, based most commonly on polymerase chain reaction (PCR), has largely replaced conventional methods owing to better overall performance, and clinical guidelines now recommend the use of these assays for CMV load monitoring in SOT and HSCT recipients to prevent or to manage CMV replication and disease [4, 5, 7, 8].

One central issue that has emerged with the use of different CMV PCR tests is the significant interassay quantification variability, as demonstrated in multicenter studies with standardized panels [9, 10]. This lack of assay agreement complicates the management of individual patients who may have testing performed in different laboratories and it has hampered the establishment of broadly applicable quantitative cutoff values that can be used in clinical decision making, potentially negatively impacting the management and long-term outcome of patients at risk of the direct and indirect effects of CMV replication [1, 4, 5]. Therefore, in the clinical management of CMV after transplantation, there is a significant unmet medical need for the development of standardized nucleic acid tests that deliver comparable quantitative data across different laboratories.

The first international standard for CMV QNAT has recently been established by the World Health Organization (WHO) Expert Committee on Biological Standardization [11]. This CMV standard should help to improve interassay agreement. However, assay-specific variability is still expected owing to underlying differences in test constituents, including varying nucleic acid extraction methods, target-specific amplification efficiencies, assay biochemistries, and operator-dependent variability. As recently suggested [12], these residual quantification disparities could be solved through the widespread availability of commercial PCR tests that encompass all assay steps (nucleic acid preparation, reaction setup, calibration, amplification, and detection) and demonstrate reliable interlaboratory quantification as defined by agreement and precision. Here, we report the results of a multicenter international study designed to determine the comparability of quantitative data and precision of a new, fully automated, Food and Drug Administration–approved CMV QNAT (COBAS AmpliPrep/COBAS TaqMan CMV Test [CAP/CTM CMV test]) using a blinded panel across diverse laboratories and to compare these 2 parameters among the 5 different assays currently used in these laboratories. Agreement between the CAP/CTM CMV test and the diverse in-house quantification assays was also defined using clinical plasma specimens from immunocompromised individuals.

## METHODS

### CAP/CTM CMV Test Colinearity to the First WHO CMV International Standard

The CAP/CTM CMV test (Roche Molecular Systems, Inc [RMS], Branchburg, New Jersey) analytical performance characteristics, including traceability to the first WHO CMV international standard for nucleic acid amplification techniques (NIBSC 09/162) were described previously [13]. In brief, the CAP/CTM CMV test uses primers and probes targeting a conserved region of the CMV genome (UL54, virus encoded DNA polymerase) and has a linear quantification range from 150 to 10 000 000 (2.18–7.0 log_10_) copies/mL representing 137 and 9 100 000 (2.14–6.96 log_10_) IU/mL, respectively (1 copy = 0.91 IU). Several standards and control specimens were used during the development of the test to achieve traceability to the first WHO CMV international standard as recommended by the Clinical and Laboratory Standards Institute guidelines [14]. The standards included the WHO CMV standard, RMS CMV secondary standard, RMS CMV secondary standard source material (CMV strain AD169), and RMS CMV calibration panel (Lambda CMA1.2). The standards, the calibration panel, and an independent CMV clinical specimen were tested at similar levels to determine whether colinearity to the WHO CMV standard was achieved. Assessment of colinearity was performed to demonstrate that the WHO standard was commutable at any given titer throughout the measuring range and to thereby ensure traceability. The concentration range tested for the WHO CMV standard was from 500 IU/mL to 50 000 IU/mL (2.70–5.70 log_10_ IU/mL), the RMS CMV secondary standard source material was tested from 500 IU/mL to 10 million IU/mL (2.70–7.00 log_10_ IU/mL), the RMS CMV calibration panel was tested from 523 to 9.3 million IU/mL (2.72–6.97 log_10_ IU/mL), and the independent CMV clinical specimen was tested from 500 IU/mL to 22 686 IU/mL (2.70–4.36 log_10_ IU/mL). The standard and control specimens were demonstrated to be colinearly distributed to the WHO material across the linear range of the CAP/CTM CMV test (Supplementary Figure 1).

### CMV DNA Quantification Tests

CMV DNA quantification with the CAP/CTM CMV test was compared to in-house tests of record at each of 5 academic centers including The John Hopkins Hospital (site 2, real-time PCR based on Artus reagents [Qiagen, Germantown, Maryland]), Hospital Universitario de la Princesa (site 3, Affigene real-time PCR test [Cepheid, Sunnyvale, California]) University of Basel (site 4, user-defined real-time PCR [*UL111a* gene target]), Stanford University (site 5, COBAS AMPLICOR MONITOR CMV test [RMS]), and Helsinki University Hospital (site 6, user-defined real-time PCR [pp65 gene target]). The analytical performance characteristics for commercial and laboratory-developed quantitative PCR assays have been described previously [15–19].

### Comparability and Reproducibility of the CAP/CTM CMV Test and 5 Quantitative PCR Assays

Comparability and reproducibility of the CAP/CTM CMV test was studied in comparison with 3 assays based on commercial reagents, and 2 tests that use laboratory-developed primers and probes using a panel prepared from a well-characterized CMV cultured virus stock (strain AD-169, titer assigned by the COBAS AMPLICOR CMV MONITOR Test). The panel consisted of 6 dilutions; 150, 550, 2000, 20 000, 50 000, and 5 000 000 copies/mL (2.18, 2.74, 3.3, 4.3, 4.7, and 6.7 log_10_ copies/mL, respectively). These dilutions covered the dynamic range of the CAP/CTM CMV test and represent relevant clinical viral load thresholds [1–7, 13]. The prepared virus stock dilutions were further diluted in CMV-negative human ethylenediaminetetraacetic acid plasma. Each site tested 15 replicates of each panel member with CAP/CTM CMV and in-house tests except site 2 (15 replicates by CAP/CTM CMV/12 replicates of in-house test). The CMV DNA panel was prepared at RMS and shipped to the study sites labeled with coded sample identification numbers to ensure that the site study staff was blinded to the CMV DNA concentration of each panel member. These experiments were designed in accordance with guidelines for establishing analytical performance characteristics of QNATs [20].

### Quantitative Agreement Between CAP/CTM CMV Test and 5 Quantitative PCR Assays Using Clinical Specimens From Immunocompromised Patients

Agreement between CAP/CTM CMV test and the 5 quantitative PCR assays described above was investigated with plasma samples collected at the study sites from immunocompromised patients monitored for CMV replication and disease. Specimens were assayed only at the site that performed original testing. In addition, 403 samples from 135 HSCT recipients participating in the maribavir prophylaxis for prevention of CMV phase 3 trial (NCT00411645) were provided by ViroPharma, Inc, to the study sites for PCR testing [21]. Only patients with plasma samples with a volume >600 μL were included in this analysis, and due to volume requirements for in-house testing, site 2 did not quantify samples from the maribavir prophylaxis trial. Plasma samples from the maribavir prevention trial were randomly distributed to the other 4 study sites. Institutional review board approval was obtained at each institution for this study.

### Statistical Analysis

The precision of log_10_-transformed valid test results within the linear range of each assay was estimated at each expected log_10_ CMV DNA concentration. The log-normal mean and log-normal coefficient of variation (%) and 95% confidence intervals (CIs) (including the lower and upper confidence limits) for total variance were calculated using the linear mixed effect model with site and day/run, and within-run as random effects.

Deming regression analysis of the viral load results for each local assay vs the CAP/CTM CMV test was performed to evaluate the correlation between the assays overall and by study site. All the statistical analyses were performed using the statistical software SAS version 9.2.

## RESULTS

### Comparability and Reproducibility of Quantitative PCR Assays With a Standardized CMV DNA Panel

**Figure 1.**
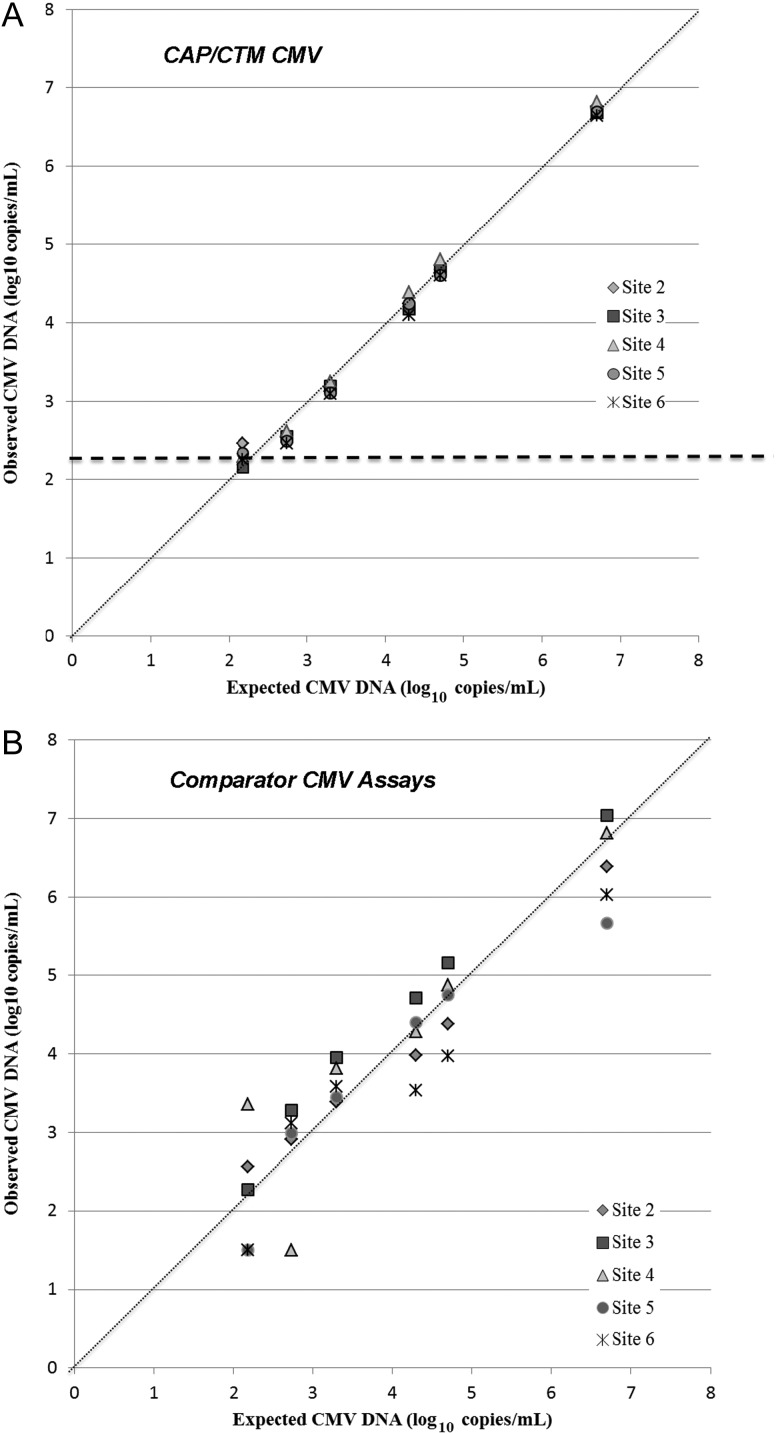
Comparability of quantitative data across laboratory sites. A dilution series of cytomegalovirus (CMV) AD-169 was prepared using cytomegalovirus-seronegative plasma. Geometric means of tested replicates are plotted. Numerical ranges indicate 95% confidence intervals of the means of each panel member at each site. *A*, COBAS AmpliPrep/COBAS TaqMan CMV data. Dashed line indicates assay lower limit of quantification. *B*, Data from 5 comparator polymerase chain reaction assays. Abbreviations: CAP/CTM CMV, COBAS AmpliPrep/COBAS TaqMan CMV Test; CMV, cytomegalovirus.

To determine comparability of quantitative data obtained with CAP/CTM CMV test across laboratories vs participating centers' PCR assays, plasma panels spiked with CMV strain AD-169 from 2.18–6.7 log_10_ copies/mL were tested at the 5 study sites. The level of quantitative agreement was high for the CAP/CTM CMV test across the different laboratories as demonstrated by a smaller range of mean concentrations of panel members by site and narrower CIs of the combined data per panel member compared to in-house PCR test in clinical use at the study sites (Figure 1). For CAP/CTM CMV, the greatest quantitative variability was observed for the lowest concentration panel member (2.18 log_10_ copies/mL, the test's lower limit of quantification); all replicates were detected, but 62/75 (83%) could not be quantified. This panel member was also variably detected by each comparator PCR assay. The AMPLICOR assay (site 5) failed to detect 11/15 replicates (73%), whereas the Affigene CMV trender test (site 3) was able to quantify 15/15 replicates. Overall, of 72 valid comparator PCR assay results, 12 replicates were not detected, 22 were below the lower limit of quantification, and 38 (53%) were quantified (Supplementary Table 1).

**Figure 2.**
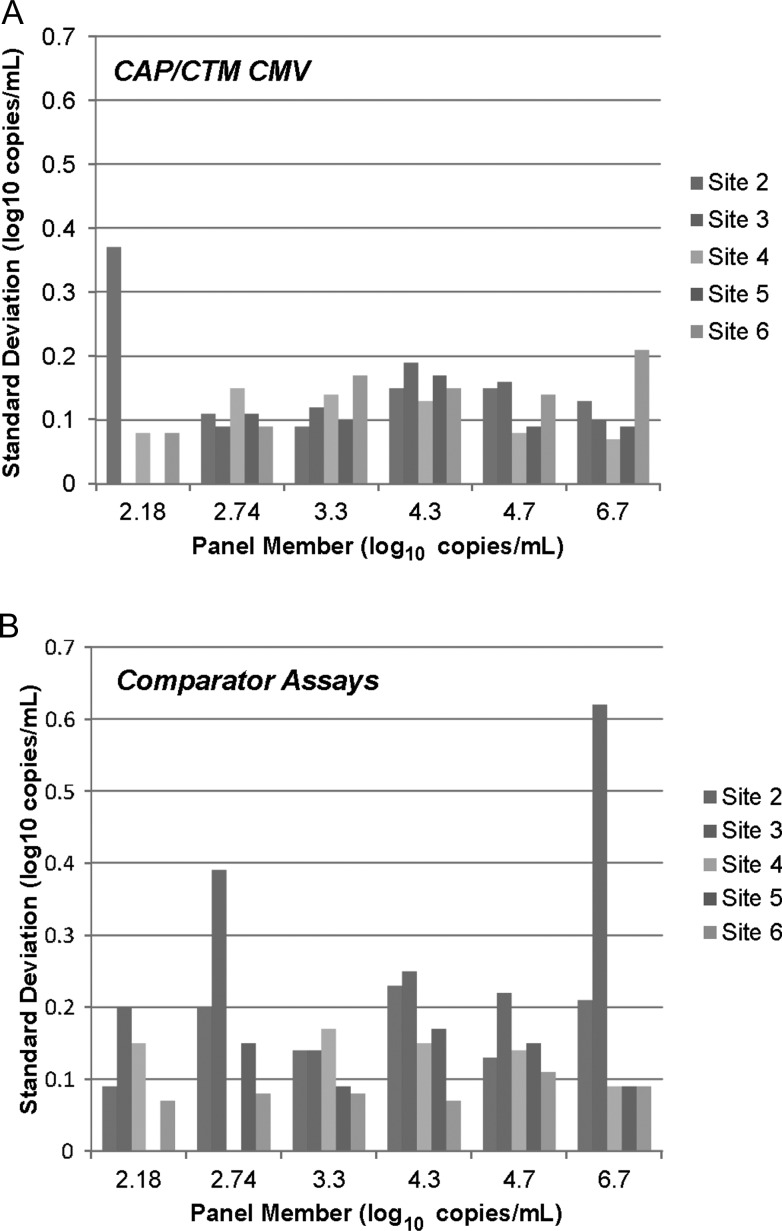
Reproducibility of quantitative data across laboratory sites. Standard deviations of replicates from cytomegalovirus (CMV) AD-169 dilution series are plotted. *A*, COBAS AmpliPrep/COBAS TaqMan CMV Test precision by study site. Quantifiable replicates at 2.18_log10_ copies/mL: site 2, n = 2; site 3, n = 0; site 4, n = 6; site 5, n = 1; site 6, n = 4. Standard deviations at 2.18_log10_ copies/mL were not calculated for sites 3 and 5. At 2.74_log10_ copies/mL, all replicates were quantifiable at all sites except site 6 (11/15 quantifiable). *B*, Precision for the 5 comparator polymerase chain reaction assays. Replicates of each panel member tested at site 2, n = 12; at sites 3–6, n = 15. Quantifiable replicates at 2.18_log10_ copies/mL: site 2, n = 2; site 3, n = 15; site 4, n = 11; site 5, n = 0; site 6, n = 10. Standard deviation at 2.18_log10_ copies/mL was not calculated for site 3. At 2.74_log10_ copies/mL, all replicates were quantifiable at all sites except site 4 (0/15 quantifiable) and site 5 (9/15 quantifiable). Abbreviation: CAP/CTM CMV, COBAS AmpliPrep/COBAS TaqMan CMV Test.

Data from experiments with the spiked plasma panel were also used to compare the precision of the CAP/CTM CMV test to the in-house PCR assays used by the study sites. The standard deviation was <0.2 log_10_ copies/mL for most panel members across the different sites performing the CAP/CTM CMV test, with 2 exceptions (2.18 and 6.7 log_10_ copies/mL for site 2 and site 6 tests, respectively, Figure 2). Similar reproducibility was observed for the AMPLICOR test (site 5), CMV real-time PCR (UL111a PCR, site 4) and real-time PCR (pp65 PCR, site 6) in-house assays. The Artus CMV PCR (site 2) and Affigene CMV (site 3) trender tests demonstrated greater imprecision (standard deviations >0.2 log_10_ copies/mL for multiple panel members). Coefficients of variation demonstrated similar trends (Supplementary Figure 2).

### Quantitative Agreement Between CAP/CTM CMV Test and 5 Quantitative PCR Assays Using Clinical Specimens From Immunocompromised Patients

**Figure 3.**
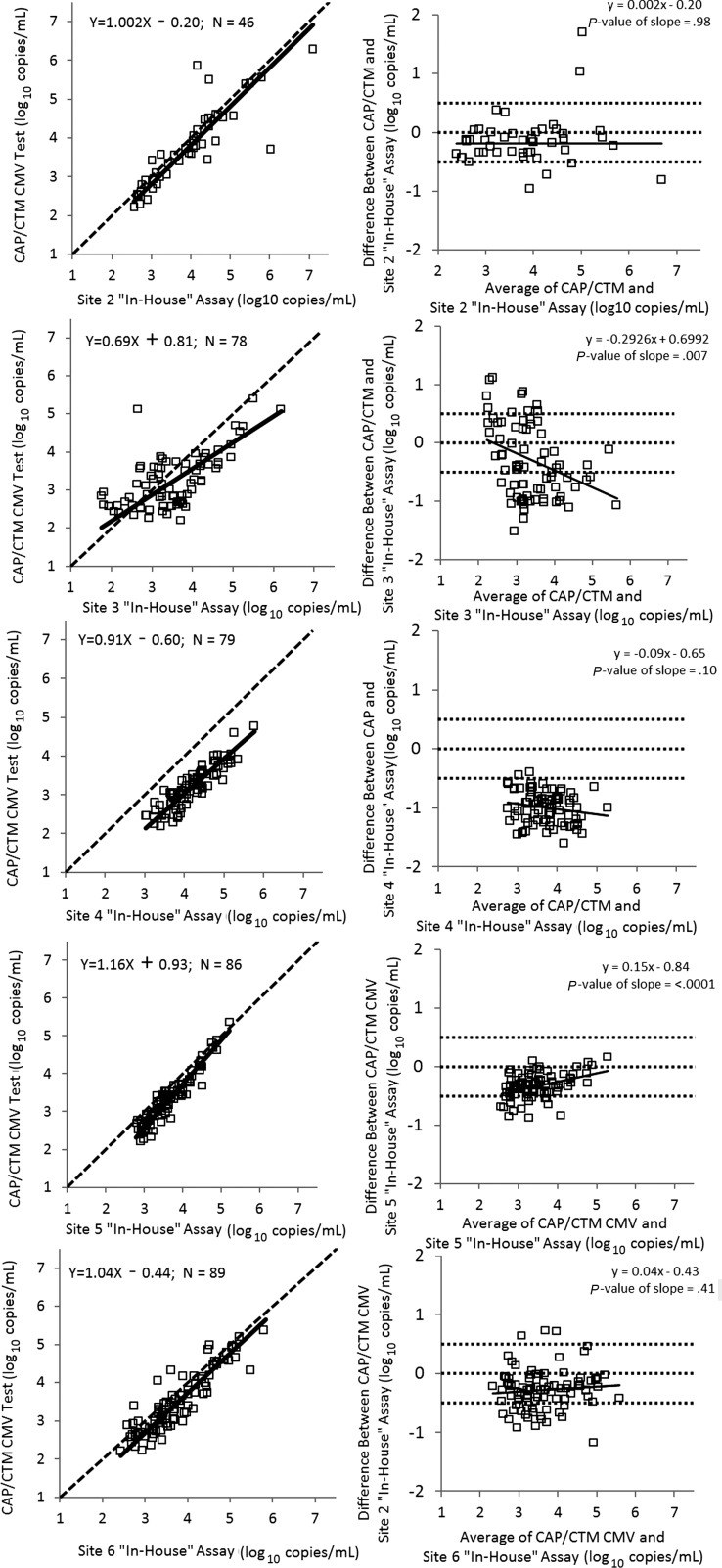
Agreement in cytomegalovirus (CMV) DNA load measurement between the COBAS AmpliPrep/COBAS TaqMan CMV Test (CAP/CTM CMV) and 5 comparator polymerase chain reaction (PCR) assays in plasma samples from immunocompromised individuals. Left-hand panels, agreement plots with Deming regression lines; dashed line indicates 100% agreement level. Right-hand panels, Bland-Altman plots (difference in quantification between the CAP/CTM CMV test and comparator PCR assays vs mean of the 2 measurements). Solid line, least squares regression; dashed lines, mean differences of +0.5/0/−0.5 log_10_ copies/mL. Abbreviation: CAP/CTM CMV, COBAS AmpliPrep/COBAS TaqMan CMV Test.

Trends in quantitative disagreement between the CAP/CTM CMV test and study sites' PCR assays were defined by comparing viral loads obtained by the 2 tests on individual plasma samples from immuncompromised patients. HSCT recipients comprised 67% (267/396) of the patients studied; these individuals contributed 80% and 76% of the total number of samples and valid PCR test results, respectively (Table 1). Two patterns of disparity were observed (Figure 3). CAP/CTM CMV test yielded lower values than 3 in-house tests (sites 2, 4, and 6) throughout the measuring range. Bland-Altman analysis demonstrated this constant bias (least squares regression slope absolute value <.1, with nonsignificant *P* value). Additionally, for in-house assays at sites 3 and 5, the difference in quantification compared to the CAP/CTM CMV test varied throughout the measuring range. Bland-Altman analysis further demonstrated this proportional bias (least squares regression slope absolute value >.1, with significant *P* value, Figure 3).

**Table 1.** Patient Populations and Number of Samples Used in the Reproducibility Comparison of COBAS AmpliPrep/COBAS TaqMan CMV Test and 5 Quantitative Polymerase Chain Reaction Assays

Patient Population	Total No. of Samples Tested	No. of Valid Test Results^a^
Solid organ transplant recipients (n = 107)	107	71
Hematopoietic stem cell transplant recipients (n = 267)	531	286
HIV-infected patients (n = 9)	9	9
Other immunocompromised patients (n = 13)^b^	13	12

Abbreviation: HIV, human immunodeficiency virus.

^a^ Only samples with paired results within the linear range of the polymerase chain reaction assays were included in the comparison analysis.

^b^ Other immunocompromised patients included subjects diagnosed with hematologic malignancies (n = 9) or autoimmune diseases (n = 4) receiving immunosuppressive therapy.

## DISCUSSION

The data from this multicenter international study demonstrate that the CAP/CTM CMV test performs consistently across laboratories, from the perspective of quantitative agreement and reproducibility. Furthermore, the finding of constant and variable quantification differences among PCR assays currently used at the participating centers compared with the CAP/CTM CMV test underscores the challenges in achieving a general quantitative standardization. Constant bias between different assays likely reflects the use of different calibrators in assays that are otherwise functionally similar. In these instances, interassay agreement may improve with adoption of a calibrator based on the international standard; alternatively, a conversion factor can be applied to normalize data if the use of a different calibrator is not feasible. These fairly easy adjustments are unlikely to improve agreement between assays whose functionality is sufficiently different to result in variable quantification differences throughout the measuring range. For these assays (with variable quantification), assay traceability to the international standard alone will not adequately correct for these types of differences. Instead, these assays must also demonstrate colinearity to the international standard throughout the assay measuring range. Ideally, this approach (to calibrate and establish colinearity to the reference material, eg, the first WHO CMV international standard) should be used whenever samples are evaluated in patients who are monitored for CMV DNA as part of the management of SOT and HSCT recipients.

Monitoring CMV DNA has become critical for the early identification of viral replication for preventing progression to disease in the posttransplant period, and for monitoring the response to antiviral treatment in patients with CMV replication and disease. In SOT, universal prophylaxis and preemptive treatment approaches are both used to prevent CMV disease [1, 2, 4, 5]. Although both approaches are currently viewed as equivalent, the advantages of prophylaxis are prevention of replication and disease in the immediate posttransplant period and elimination of the need for viral load monitoring during prophylaxis, particularly for the high-risk, CMV-seropositive donor/CMV-seronegative recipient SOT. However adverse events associated with prolonged antiviral drug administration have limited the utility of this approach in some patients. Also, viral load monitoring may still be useful in some patients in whom drug-resistant viruses are suspected to emerge when treated with lower antiviral doses in an attempt to mitigate drug side effects. Finally, the onset of late CMV disease has been observed in up to 29% of SOT recipients after prophylaxis cessation [1, 2, 4].

Viral load monitoring is the key feature of preemptive therapy. In this alternate approach, antiviral therapy is initiated before the onset of CMV disease when viral load measurements reach a predictive threshold. Advantages of preemptive therapy include a smaller proportion of treated patients, shortened therapeutic duration, lowered costs associated with posttransplant medications, and reduced occurrence of drug toxicity (primarily bone marrow suppression). Avoidance of marrow suppression is the major rationale for the use of preemptive strategies in allogeneic HSCT in the preengraftment period. Disadvantages of the preemptive antiviral strategy include risk of disease prior to treatment initiation in individuals with fast replication, indirect effects of CMV replication (in the absence of disease) on allograft survival and mortality, and laboratory costs due to more frequent viral load monitoring. Perhaps one of the most significant drawbacks to this approach is the lack of generally established quantitative cutoffs that are predictive of CMV disease in recipients of different allografts due in large part to the lack of standardized quantification assays that perform comparably across laboratories.

The lack of standardized CMV load assays has also complicated CMV management in other ways. For individual patients, quantification disparities across laboratories dictate that a single laboratory should be used for viral load testing, so that results can be accurately interpreted. In addition, although higher viral loads have been shown to correlate with an increased risk of disease in SOT and HSCT patients [1, 4, 5], the lack of standardization has hampered the development of discrete, globally applicable, quantitative predictors of active disease, and other cutoffs that can be used to determine relapse risk and adequate treatment duration [12]. Currently, the burden of defining these cutoffs is placed on individual laboratories, and as a result, clinically relevant values still vary from center to center.

The implementation of an international standard and the availability of commercial QNATs with broad interlaboratory agreement that are traceable and colinear to the first WHO CMV international standard represent a much-needed advancement. As demonstrated here for CAP/CTM CMV, precise, accurate, and standardized results should allow the design of multicenter studies to delineate testing algorithms, including quantitative cutoffs and testing frequencies that enhance clinical outcomes of CMV infections in HSCT and SOT patients. In turn, these data can be used as the basis for management guidelines that should significantly clarify decision making for clinicians and improve infection outcomes in at-risk patients.

## Supplementary Data

Supplementary materials are available at *Clinical Infectious Diseases* online (http://www.oxfordjournals.org/our_journals/cid/). Supplementary materials consist of data provided by the author that are published to benefit the reader. The posted materials are not copyedited. The contents of all supplementary data are the sole responsibility of the authors. Questions or messages regarding errors should be addressed to the author.

Supplementary Data
