# Supplementary material for: An International Multicenter Performance Analysis of Cytomegalovirus Load Tests
Source: Clin Infect Dis. 2012 Oct 24;56(3):367–73. doi: 10.1093/cid/cis900 (PMC3540041; doi:10.1093/cid/cis900)
Supplement: Supplementary Data [file supp_56_3_367__index.html]

An International Multicenter Performance Analysis of CMV Viral Load Tests — An International Multicenter Performance Analysis of Cytomegalovirus Load Tests — An International Multicenter Performance Analysis of Cytomegalovirus Load Tests — Supplementary Data 

# An International Multicenter Performance Analysis of Cytomegalovirus Load Tests

## Supplementary Data

Supplementary Data

**Files in this Data Supplement:**

- Supplementary Data - Doc file
