# Supplementary material for: An International Multicenter Performance Analysis of Cytomegalovirus Load Tests
Source: Clin Infect Dis. 2012 Oct 24;56(3):367–73. doi: 10.1093/cid/cis900 (PMC3540041; doi:10.1093/cid/cis900)
Supplement: Supplementary Data [file supp_cis900_cis900supp.doc]

**Supplemental Table 1:** Tested and Detected Replicates, CAP/CTM CMV vs. Comparator PCR Assays^#^

| **PCR**  **Assay** | **Panel Member (log10 copies/mL)** | **Site** | **No. of Replicates Tested** | **No. of Valid Tests** | **Target Not Detected** | **< LLOQ** | **Within Assay Range** |
| --- | --- | --- | --- | --- | --- | --- | --- |
| CAP/CTM CMV | 2.18 | 2 | 15 | 15 | 0 | 13 | 2 |
|  |  | 3 | 15 | 15 | 0 | 15 | 0 |
|  |  | 4 | 15 | 15 | 0 | 9 | 6 |
|  |  | 5 | 15 | 15 | 0 | 14 | 1 |
|  |  | 6 | 15 | 15 | 0 | 11 | 4 |
| Comparator PCR assays | 2.18 | 2 | 12 | 12 | 0 | 10 | 2 |
|  |  | 3 | 15 | 15 | 0 | 0 | 15 |
|  |  | 4 | 15 | 15 | 1 | 3 | 11 |
|  |  | 5 | 15 | 15 | 11 | 4 | 0 |
|  |  | 6 | 15 | 15 | 0 | 5 | 10 |
| CAP/CTM CMV | 2.74 | 2 | 15 | 15 | 0 | 0 | 15 |
|  |  | 3 | 15 | 15 | 0 | 0 | 15 |
|  |  | 4 | 15 | 15 | 0 | 0 | 15 |
|  |  | 5 | 15 | 15 | 0 | 0 | 15 |
|  |  | 6 | 15 | 15 | 0 | 4 | 11 |
| Comparator PCR assays | 2.74 | 2 | 12 | 12 | 0 | 0 | 12 |
|  |  | 3 | 15 | 15 | 0 | 0 | 15 |
|  |  | 4 | 15 | 15 | 3 | 12 | 0 |
|  |  | 5 | 15 | 15 | 3 | 3 | 9 |
|  |  | 6 | 15 | 15 | 0 | 0 | 15 |

#Data for two lowest concentration panel members are shown; all replicates for all panel members >2.74 log_10_ copies/mL were within the assays’ measuring ranges.

Abbreviations: LLOQ, lower limit of quantification; ULOQ, upper limit of quantification.

**Supplemental Figure Legends**

**Figure 1:** Test for co-linearity between 1st WHO International Standard and Standards used in calibration and standardization of the CAP/CTM CMV Test.


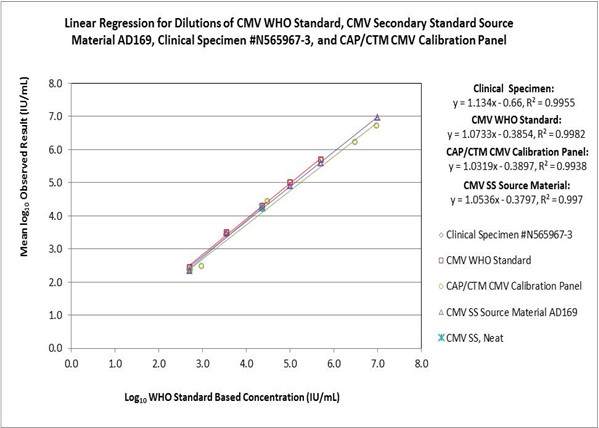


**Figure 2: Reproducibility of quantitative data across laboratory sites.** Percent Coefficient of Variation (% C.V.) from CMV AD-169 dilution panel is plotted. (A) CAP/CTM CMV test variability by study site. (B) Precision for the five comparator PCR assays.

**
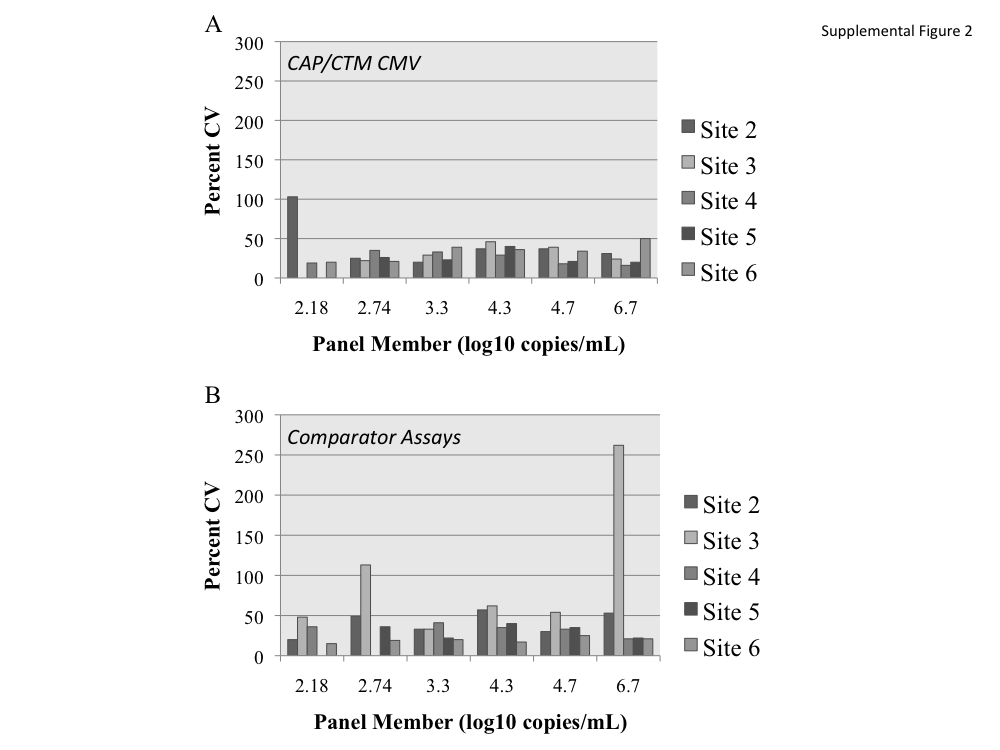
**
